# Soft Sarcomatous Tumor of the Antrum of Highmore

**Published:** 1865-12

**Authors:** 


### Selections.

### SOFT SARCOMATOUS TUMOR OF THE ANTRUM OF HIGHMORE.

At the meeting of the Pathological Society, November 8th, Dr. Sands in the chair,

Dr. Krakowitzer related an interesting case of tumor of the antrum, of which we give the substance.

The patient was a young man, 25—26 years of age, who six years ago had been an inmate of the Jews hospital. He was then laboring under ozena, a discharge taking place from both nostrils, which were ulcerated and covered with crusts.

The treatment consisted in removing the crusts, cauterization of the ulcerated surfaces, and the use of mild, astringent injections. In the minutes of the case it was stated that he had had hæmoptysis, and that a severe attack of bleeding had followed after probing the nasal passages. There were no hereditary taints traceable.

During the last year he had been engaged as a conductor on a railroad. One night in December last, while following this avocation, he stepped from a warm car upon a platform and unbuttoned his coat. Three days afterward he was seized with violent bleeding from the left nostril. The hæmorrhage stopped, but from that time he commenced to notice difficulty of breathing through this nostril, which difficulty gradually increased until about three months ago, when the passage had become entirely impervious, and the patient by introducing his little finger could feel a tumor descending down the nostril. In July, about six months after the first, another severe hæmorrhage occurred, upon which the tumor receded, and he could feel it no longer in the nostril. Soon, however, a swelling made its appearance on the inner canthus of the left eye, increasing until it pushed the eyeball outward to such an extent that the eyelids barely covered the eyeball, which, however, was movable, and the eyesight remaining good. The patient at this time had settled at Warrenton, Va.

Dr. Krakowitzer saw the patient a week ago. He seemed to be in perfect health, felt perfectly well, and had never suffered any pain from the swelling. The whole of the left side of the face is a little protruding. In consequence of the bulging out of the eyeball and pressure upon the lachrymal apparatus, there was constant epiphora. On examining the left superior maxilla, it gave to the touch a sensation of softness and elasticity. No swelling could be detected in the nose. The soft palate was normal. There was no affection of the submaxillary or of the cervical glands. On pressure the inferior margin of the orbit could be felt intact. Since his arrival in New York the patient has had three attacks of hæmorrhage from the nasal passages, of considerable severity.

There could be no doubt of the presence of a tumor. In regard to its character the opinion was formed that the new formation was not malignant. Its growth had evidently not been very fast, and there was an absence of pain and no infiltration of the neighboring glands.

The next point to be decided was regarding the origin of the disease. The patient stated distinctly that the tumor at first presented in the nasal cavity, and that with its recession from that locality it could be detected in the angle of the eye. The general aspect of the face and the soft elastic feel pointed to the antrum of Highmore as the seat of the disease.—The first development probably took place toward the nose; then, with its advancing growth becoming disengaged, it was lifted back, toward the orbit, and the anterior walls of the antrum became gradually absorbed. It was presumed that there were no adhesions with the base of the scull.

The removal of the tumor was decided upon, and the operation performed in the usual manner of extirpating tumors involving these parts. The whole of the antrum was found filled with the mass. In removing the soft fragments of the growth extending toward the base of the skull, it was found that *bony absorption had taken place here*, and at one point so completely that the *dura mater was visible*, and the pulsations of the brain could be seen.

The tumor, as had been anticipated, belonged to the soft sarcomatous variety, not exactly malignant, but still apt to recur, especially in this region. The prognosis in this case is that the patient will die of meningitis.

Under the miscroscope the main characteristic element of the tumor, which on account of its softness could not be removed *en masse*, but had to be taken away in fragments, consisted of elongated cells with one single nucleus.

